# Determinants of Treatment Adherence Among Smear-Positive Pulmonary Tuberculosis Patients in Southern Ethiopia

**DOI:** 10.1371/journal.pmed.0040037

**Published:** 2007-02-13

**Authors:** Estifanos Biru Shargie, Bernt Lindtjørn

**Affiliations:** 1 Centre for International Health, University of Bergen, Bergen, Norway; 2 Southern Nations, Nationalities and Peoples' Regional State Health Bureau, Awassa, Ethiopia; University of California San Francisco, United States of America

## Abstract

**Background:**

Defaulting from treatment remains a challenge for most tuberculosis control programmes. It may increase the risk of drug resistance, relapse, death, and prolonged infectiousness. The aim of this study was to determine factors predicting treatment adherence among smear-positive pulmonary tuberculosis patients.

**Methods and Findings:**

A cohort of smear-positive tuberculosis patients diagnosed and registered in Hossana Hospital in southern Ethiopia from 1 September 2002 to 30 April 2004 were prospectively included. Using a structured questionnaire, potential predictor factors for defaulting from treatment were recorded at the beginning of treatment, and patients were followed up until the end of treatment. Default incidence rate was calculated and compared among preregistered risk factors. Of the 404 patients registered for treatment, 81 (20%) defaulted from treatment. A total of 91% (74 of 81) of treatment interruptions occurred during the continuation phase of treatment. On a Cox regression model, distance from home to treatment centre (hazard ratio [HR] = 2.97; *p* < 0.001), age > 25 y (HR = 1.71; *p* = 0.02), and necessity to use public transport to get to a treatment centre (HR = 1.59; *p* = 0.06) were found to be independently associated with defaulting from treatment.

**Conclusions:**

Defaulting due to treatment noncompletion in this study setting is high, and the main determinants appear to be factors related to physical access to a treatment centre. The continuation phase of treatment is the most crucial time for treatment interruption, and future interventions should take this factor into consideration.

## Abstract 2

A prospective cohort study of smear-positive tuberculosis patients at an Ethiopian hospital found treatment default rates to be high; the main factors relate to physical access to the treatment centre.

## Editors' Summary

**Background.:**

Tuberculosis (TB) is one of the leading causes of death from infectious disease worldwide, and it kills around 1.7 million people each year. TB can be successfully treated but the treatment course is long (at least six months). In 1995 the World Health Organization set up “DOTS”, an international strategy for TB control. One of the links below explains what DOTS is in more detail. One of the main elements of DOTS involves the use of standard courses of drug treatment, with the recommendation that trained observers watch people take their treatment. These steps should prevent people from failing to complete their course of treatment, and the World Health Organization has set a target level of 85% for treatment success. However, people do often have problems sticking to treatment and the reasons for this are not clearly understood. Factors such as access to care and a person's social and financial situation might affect whether an individual sticks to their prescribed treatment.

**Why Was This Study Done?:**

This study was carried out in Ethiopia, which has been recognized as being in the top 22 countries with the highest burden of tuberculosis. In the region of southern Ethiopia studied, the proportion of patients not completing their treatment has declined from 38% to 18% between 1994 and 2000. However, the World Health Organization's targets have not been met, with 20% of patients currently failing to complete treatment. These researchers wanted, therefore, to identify the factors that play a part in determining whether a patient completes their course of treatment. They hope that once such factors are identified, they could ultimately be overcome with appropriate interventions and the level of treatment success improved.

**What Did the Researchers Do and Find?:**

The researchers carried out a cohort study, in which all patients diagnosed with clinical tuberculosis during a particular period of time at a major regional hospital in southern Ethiopia were included. Patients were followed up throughout their treatment course and then counted as either having defaulted from treatment (if they had been on treatment for at least 4 weeks and then interrupted for at least 8 weeks), or having completed. The researchers carried out interviews with each participant at the start of the study, to collect information on factors which might affect how each participant might stick to their treatment plan. These factors included basic information such as whether the patient was male or female, their age, marital status, educational level and occupation, as well as others including family income, whether their home was rural or urban, and the distance to the treatment centre. Finally, patients who stopped their treatment were asked an open-ended question: “Why did you stop taking TB medication?”

404 patients were included in the study and 20% defaulted, most of these within the first few months of treatment. The researchers found that a number of factors seemed to be linked to an increased chance that the person would default from treatment. These included age (patients over 25 were less likely to complete); living in a rural setting; having a lower level of education; greater distances from home to the treatment centre; the need for transport to get to treatment; and whether the patient was admitted to hospital in a serious condition. When defaulters were questioned about the reasons they did not complete treatment, the main reasons were related to physical access to the treatment clinic—for example that it was too far, they could not afford to get there, or were not able to walk to get treatment.

**What Do These Findings Mean?:**

The proportion of patients failing to complete their tuberculosis treatment here supports the view that the default rate in Ethiopia has been falling over the past two decades; but that it is still higher than that recommended by the World Health Organization. The researchers also found that physical access to treatment poses a significant barrier to completing treatment. In this study 72% of patients were within two hours' walk of the health facility, a much greater proportion than is the case for the general population in that region. These findings suggest that government initiatives will be needed in order to address the problem of access to treatment and therefore improve adherence. New initiatives are underway in Ethiopia to train health service workers who can provide community-based care.

**Additional Information:**

- World Health Organization (WHO) fact sheet on tuberculosis. More detailed information from WHO is available on DOTS, including the five key elements of DOTS
- The US Centers for Disease Control and Prevention has a minisite dedicated to tuberculosis, including a questions-and-answers page
- The Stop TB Partnership was established in 2000 to realize the goal of eliminating TB as a public health problem and, ultimately, to obtain a world free of TB. It comprises a network of international organizations, countries, donors from the public and private sectors, governmental and nongovernmental organizations, and individuals that have expressed an interest in working together to achieve this goal
- Médecins Sans Frontières, an international medical humanitarian organization, has information on its website about its activities in Ethiopia

## Introduction

As much as untreated tuberculosis (TB) threatens the well being of an individual and society, defaulting from treatment may increase the risk of drug resistance, relapse, and death, and may prolong infectiousness [1–3]. In resource-constrained settings where the health care services are not well developed, delayed presentation for treatment and defaulting from treatment are the two major challenges that TB programmes face [4]. The idea of direct observation of treatment (DOT) evolved from the need to improve treatment adherence. However, the role of DOT in maintaining treatment adherence appears to be surrounded by controversies. Reports from many countries favour DOT as a key component in the Directly Observed Treatment—Short Course (DOTS) strategy, a global strategy recommended by the World Health Organization for the prevention and control of TB [5–7]. Conversely, some randomised controlled studies have failed to establish the superiority of DOT over the conventional nonobserved treatment in improving treatment adherence [8,9]. As such, ensuring successful treatment completion might require addressing multiple factors beyond simple supervision of drug intake [10–14].

TB continues to be a major public health problem in Ethiopia, which ranks seventh by estimated number of cases among the 22 TB high-burden countries [15]. Before the introduction of DOTS in Ethiopia, 82% of TB patients were reported to have failed to complete treatment [16], while defaulting from DOTS in a southeastern part of the country was reported to be as low as 11.3% [17]. In the southern region of Ethiopia (Southern Nations Nationalities and Peoples' Regional State), DOTS was introduced in 1996. An earlier study on the impact of DOTS in the region reported a significantly declining trend in treatment noncompletion from 38% to 18% over six years during 1994–2000 [18]. However, one in five patients still continued to default from treatment, and its predictors are not well understood. Apart from physical access, such factors as socioeconomic status, educational level, and patient satisfaction with the care provided may influence treatment adherence. In an effort to reach the global target of 85% treatment success, it was compelling to identify, describe, and deal with factors determining treatment adherence. The current study, therefore, was aimed at determining factors that predict treatment adherence among the most infectious form of TB, smear-positive pulmonary TB (PTB+) patients.

## Methods

### Setting

This study was conducted among smear-positive TB patients diagnosed at Hossana Zonal Hospital in the Hadiya zone of the southern region of Ethiopia. The hospital serves as a referral centre for the population of Hadiya and three adjacent zones (approximately 2.5–3 million people). The health facilities within these zones register and treat all diagnosed TB patients under the DOTS. All diagnosed TB cases are registered at the unit registers, compiled at the district registers, and reported to the regional TB programme. Once diagnosed with TB, patients are provided with anti-TB medication and care free of charge. In line with the national TB and leprosy control programme guidelines [19], all new cases of TB are treated with short-course chemotherapy comprising two months on rifampicin, isoniazid, and pyrazinamide (RHZ) plus ethambutol (E) or streptomycin (S) during the initial phase of treatment. During this intensive phase, health care providers directly observe patients while they are taking daily treatment. This phase is followed by a continuation phase of six months on EH, during which time drugs for self-administered treatment are to be collected every month. Retreatment cases (return after default, relapse, or treatment failure cases) are treated with SERHZ for two months, followed by one month on ERHZ, and five months on ERH. Fixed dose combinations are used throughout the treatment period. Most TB cases are treated on ambulatory basis, with the exception of a few patients requiring admission for medical reasons. Those living in rural areas far away from the diagnostic centre are referred to the nearest health facility for treatment initiation and follow-up.

### Design and Data Collection

A cohort of PTB+ patients diagnosed and registered for treatment in Hossana Hospital from 1 September 2002 to 30 April 2004 were prospectively included. All smear-positive TB cases registered over the study period were included. Using structured interview questionnaires, sociodemographic, behavioural, and environmental factors that could potentially predict defaulting from treatment were recorded at the commencement of treatment. Data were also gathered on patient–provider interactions and satisfaction with the initial reception during the diagnostic process. Family income was calculated based on reported monthly salary for paid workers, monthly income from petty trade or daily labour, and/or prices and quantity of annual agricultural production divided by twelve months. Distance was calculated both in terms of number of hours an average person has to walk to reach the health facility from residence, and in terms of actual physical distance (in km) from home to the health facility.

The interviews were conducted by two trained nurses in the hospital. Informed oral consent was obtained from each individual upon enrolment to the study. All patients were followed throughout the treatment period, and any special event on the due course of treatment as well as treatment outcomes were documented. Health care workers in the TB clinics of the respective health facilities provided treatment, followed patients, and recorded outcomes. The principal investigator and a data clerk visited each health facility and collected the data on treatment outcomes. Two trained data collectors (high school graduates) traced and interviewed the defaulters and advised them to resume treatment.

A default case was defined as PTB+ patient who has been on treatment for at least four weeks and whose treatment was interrupted for more than eight consecutive weeks [19]. Treatment success was defined as cure (smear-negative at treatment completion and on at least one previous occasion), plus treatment completion without confirmation by smear-microscopy. Default rate in each stratum of the predictor factors was calculated against total person-time of observation. All defaulters were traced and interviewed regarding the main reason for defaulting from treatment. After the interview each defaulter was encouraged to get back to treatment at the nearest health facility of choice.

We calculated the sample size for the desired effect (HR) of 2.0 with 5% level of significance and 90% power assuming that one-fourth of exposed and one-tenth of unexposed patients would default from treatment, based on an average default rate of 20% from a previous study [18].

Data were entered and analysed using SPSS for windows version 12.0.1 (SPSS, http://www.spss.com). Continuous variables were analysed using independent sample t-test. Chi^2^ tests and HRs with 95% confidence intervals (95% CIs) were used to evaluate categorical data. The independent effect of each potential predictor variable (adjusted HR) was assessed using a Cox regression model with a backward fitting algorithm. Multiple responses to open-ended questions were recoded at the end of data entry, and each response was analysed independently.

The study was approved by the Regional Committee for Medical Research Ethics in Western Norway (REK Vest) and the Ethics Committee of Southern Nations Nationalities and Peoples' Regional State Health Bureau in Ethiopia.

## Results

Figure 1 shows the flow of study participants. A total of 404 new PTB+ patients were registered for treatment between 1 September 2002 and 30 April 2004. Of these, 82% (*n* = 331) were from Hadiya zone and 43% (*n* = 174) were female patients. A total of 21% (*n* = 83) of the patients had treatment follow-up at Hossana Hospital, whereas 43% (*n* = 169) and 36% (*n* = 139) were treated at the health centres and health stations, respectively. A total of 199 patients (49%) volunteered to have HIV testing, of which 12% (25 out of 199) were positive for HIV. A total of 81 patients (20%) defaulted from treatment, while 310 (77%) successfully completed treatment; nine (2.2%) died, one had treatment failure, and three had moved outside the region and could not be traced. Table 1 presents the baseline characteristics of the study participants.

**Figure 1:**
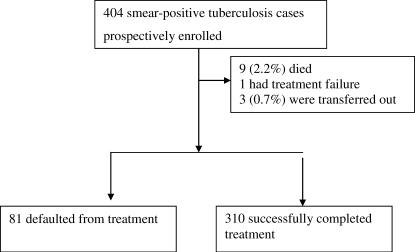
Study Profile

**Table 1:**
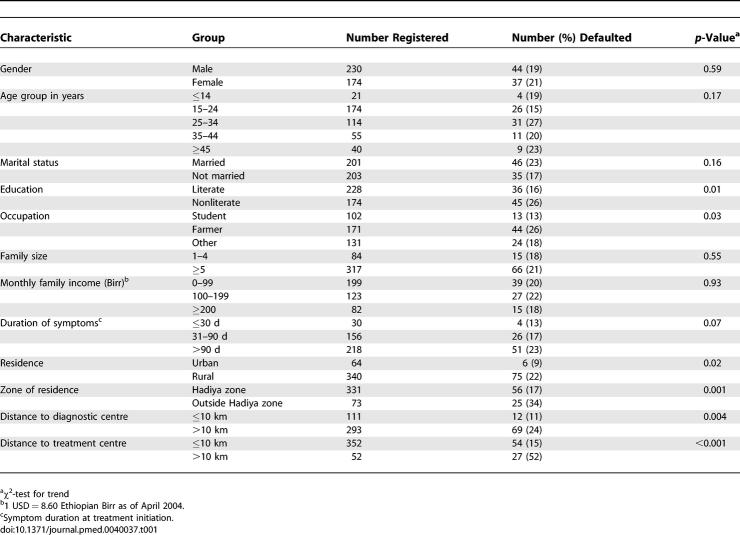
Baseline Characteristics of Smear-Positive TB Patients Registered for Treatment in Hossana Hospital from 1 September 2002 to 30 April 2004

During the first two months following the intensive phase, two-thirds of the defaulters interrupted treatment. Overall, 91% of the incidences of defaulting from treatment occurred during the continuation phase of treatment (Table 2).

**Table 2:**
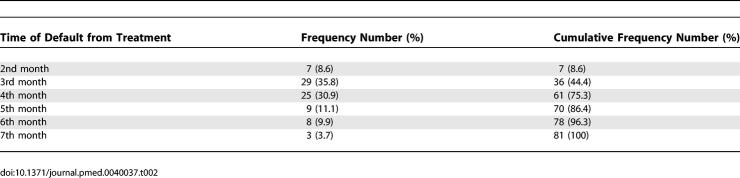
Time of Default from Treatment among the 81 Defaulters

In univariate analysis, factors significantly associated with treatment noncompletion included participant over the age of 25 y, rural residence, educational level, occupation, distance from residence to the nearest treatment centre, need for public transport to get to a treatment centre, and the patient's condition on treatment initiation, as shown in Table 3. No significant association was noted between default from treatment and gender, average family income, HIV status, treatment centre, prior knowledge about TB, and knowledge about treatment duration.

**Table 3:**
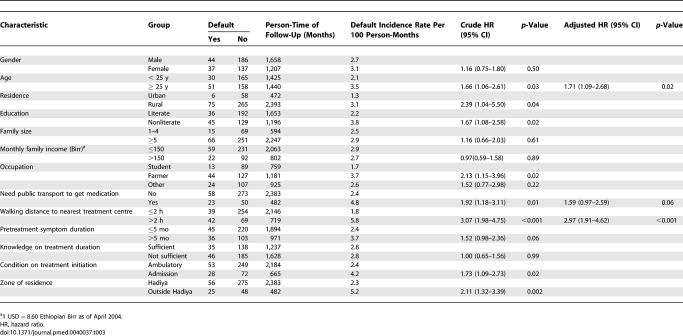
Factors Predicting Anti-TB Treatment Adherence According to Cox Regression Analysis

In multivariate analysis, three variables—age, distance from home to treatment centre, and need to use public transport to get to a treatment centre—appeared in the last step of the Cox regression model with backward elimination. Both forward and backward fittings produced the same model. After simultaneously controlling for the potential predictor variables, distance from home to treatment centre (HR = 2.97; *p* < 0.001), age over 25 y (HR = 1.71; *p* = 0.02), and necessity to use public transport to get to a treatment centre (HR = 1.59; *p* = 0.06) remained independent risk factors for treatment noncompletion (Table 3). Figure 2 shows time on treatment by walking distance from treatment centre, adjusted by Cox regression analysis.

**Figure 2:**
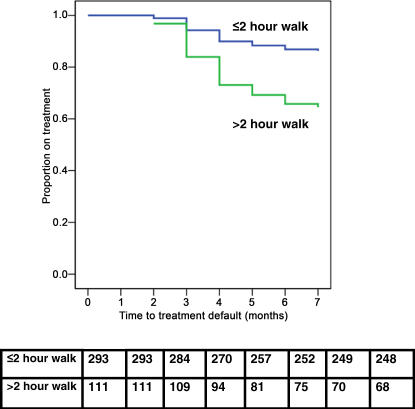
Survival Curve of Time on Treatment by Walking Distance from Treatment Centre, Adjusted by Cox Regression Analysis

It was possible to trace and advise 74 of the 81 defaulters to resume medication. A single open-ended question was posed to each defaulter case before advising them to resume treatment, “Can you tell me why you discontinued taking TB medication before the required period?” As shown in Table 4, the major reasons given by 45% of the patients for interrupting treatment were those related to physical access (TB clinic too far from home, could not afford transportation cost, and too tired to walk to the treatment centre). Erroneous sense of cure, loss of hope in the medication, and inadequate knowledge about treatment duration were also among the reasons mentioned by the patients.

**Table 4:**
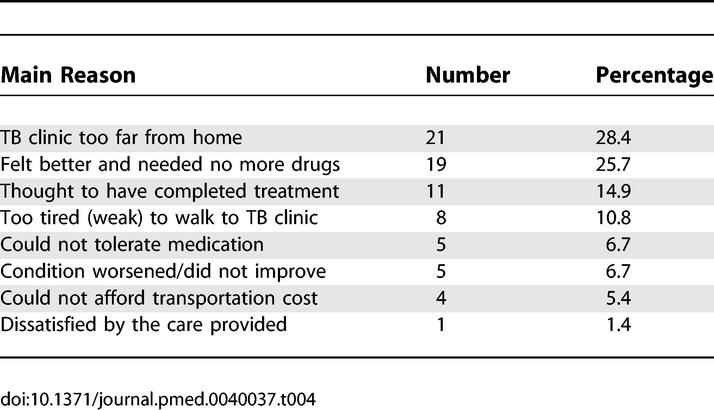
Reasons for Defaulting from Treatment (*n* = 74)

## Discussion

This study tried to measure the extent and predictors of treatment noncompletion in a predominantly rural society. A total of one-fifth of the registered PTB+ cases failed to complete treatment. This finding confirms an earlier study report that documented a declining trend in default rate from 38% to 18% over a six-year period from 1994 to 2000 [18]. The default rate in our study is lower compared to previous studies in some African countries [16, 20–22]. However, it is higher than the 11.6% default rate from DOTS reported in Arsi zone of Ethiopia [17]. Treatment noncompletion is required to fall below 10% in order to achieve treatment success of 85%, one of the health-related indicators of the Millennium Development Goals [15].

Most of the factors associated with treatment noncompletion, apart from the patient's age and level of education, are those related to physical access to health-care services: distance from home to treatment centre, rural residence, and a need to use public transport for ambulatory care. A single open-ended question to the defaulters also elucidated responses that are consistent with this finding. In other settings, risk factors such as knowledge about treatment duration, change of treatment unit, running out of drugs, poor patient–health provider communication, and medication side effects were reported to have been associated with treatment noncompletion [17, 21–25].

The prospective design of this study has allowed recording of potential risk factors for treatment noncompletion at the beginning of the study. As a result, the risk of recall bias, which is an inherent problem of retrospective study designs, is taken into account. This design would also help to predict the probability of defaulting from treatment right at the start of treatment based on the patient's sociodemographic and environmental context. However, this approach has its own limitations. For example, events during the course of treatment, such as drug side effects and care provider–patient interactions, which could considerably influence a patient's decision to continue treatment, were not measured. Such factors cannot be recorded at the beginning of the study, and a combined approach may have yielded better results. Furthermore, fewer than half of the patients volunteered for HIV screening, and as a result, we are not able to determine how much, if at all, HIV status influences treatment adherence in our study setting.

While it is recognised that only half of the population in this setting lives within reach of a health facility in a two-hour walk [26], 72% of the patients in our study cohort came from within this circle. Fewer patients came from areas not within reach of a health facility in a two-hour walk, and nearly half of these patients failed to complete treatment. As such, physical access seems to be the most significant determinant of treatment noncompletion in this predominantly rural setting, and its role in health-care seeking warrants further investigation. Age also appears to be another significant predictor for defaulting in this setting. One possible explanation is that older people assume more family responsibility and, as a result, this group of individuals may tend to stop medication when they feel better or when they come across more pressing social obligations. It is also possible that older people gain less attention in the provision of health care, and this warrants further investigation.

Although 15% of defaulters in our study mentioned that they stopped taking medication because they thought they had completed the treatment, there was no statistically significant difference between the defaulters and nondefaulters with regard to knowledge about treatment duration. There was also no difference between these groups regarding intention to discontinue treatment if and when their symptoms disappeared. However, 26% of defaulters mentioned “feeling better” as the main reason for discontinuing treatment. This indicates that distance, the strongest predictor of treatment interruption in this area, may act synergistically with other factors such as feeling better, loss of hope, unaffordable transportation costs, and drug side effects to influence adherence to treatment. Future research in this area should address the complex interplay between such factors and explore possible interventions to improve treatment adherence. Decentralisation of treatment follow-up to community health posts and improved patient–provider communication on treatment duration are among options to be explored further. Peer-encouragement mechanisms such as the formation of “TB clubs” have also been shown to be an effective community-based approach in enhancing treatment completion [27,28]. In an effort to reduce the access gap to health care, the Ethiopian government has started a new community-based initiative called the “Health Service Extension Programme.” Under this programme, thousands of health extension agents have been identified and trained, which might help to enhance case-holding under the DOTS programme through decentralisation of services in the country [29].

In this study, more than 90% of the defaulters discontinued treatment during the continuation phase of treatment, particularly during the first two months following the two-month intensive phase of treatment. One of the explanations for this finding might be that during this phase most of the symptoms disappear and patients may erroneously believe they are cured. This may encourage them to become reluctant to bear the extra burden of the cost of travel, time, and drug side effects. On the contrary, the condition may remain the same or even be worsened by such conditions as HIV/AIDS or resistance of the bacilli to anti-TB medication, and patients may lose hope for improvement after having made every effort during the first two months. A better understanding of treatment default timing in most patients may help programme managers to better plan ahead for how to reduce this problem. Furthermore, the importance of good rapport between patients and care providers in proper treatment adherence cannot be overemphasised.

Although the reported death rate in this study cohort is only 2.2%, there is a possibility of more deaths among patients who defaulted from treatment. Unfortunately, we do not have sufficient information on the final status of these defaulters after the initial contact and advice to resume treatment. Likewise, only one of 404 patients was reported to have treatment failure, which is unlikely with the current eight-month regimen used in this study setting [30]. Nearly half of smear-positive TB patients do not have a follow-up sputum examination in this set-up [18], and as a result this reported failure rate is likely to be an underestimate.

In conclusion, defaulting from treatment in this study setting was high, and the main determinants appear to be factors related to physical access to a treatment centre. The continuation phase of treatment is the most crucial time for defaulting from treatment, and future interventions should take this into consideration.

## Abbreviations

CI: confidence interval
DOT: direct observation of treatment
DOTS: Directly Observed Treatment—Short Course
HR: hazard ratio
PTB+: smear-positive pulmonary TB
TB: tuberculosis
